# Role of Neuroinflammation in Adult Neurogenesis and Alzheimer Disease: Therapeutic Approaches

**DOI:** 10.1155/2013/260925

**Published:** 2013-04-03

**Authors:** Almudena Fuster-Matanzo, María Llorens-Martín, Félix Hernández, Jesús Avila

**Affiliations:** ^1^Department of Molecular Neurobiology, Centro de Biología Molecular Severo Ochoa (CSIC-UAM), Madrid, Spain; ^2^Centro de Investigación Biomédica en Red sobre Enfermedades Neurodegenerativas (CIBERNED, ISCIII), 28031 Madrid, Spain

## Abstract

Neuroinflammation, a specialized immune response that takes place in the central nervous system, has been linked to neurodegenerative diseases, and specially, it has been considered as a hallmark of Alzheimer disease, the most common cause of dementia in the elderly nowadays. Furthermore, neuroinflammation has been demonstrated to affect important processes in the brain, such as the formation of new neurons, commonly known as adult neurogenesis. For this, many therapeutic approaches have been developed in order to avoid or mitigate the deleterious effects caused by the chronic activation of the immune response. Considering this, in this paper we revise the relationships between neuroinflammation, Alzheimer disease, and adult neurogenesis, as well as the current therapeutic approaches that have been developed in the field.

## 1. Introduction

The inflammatory response is an early, specialized immune reaction to tissue damage or pathogen invasion. In the central nervous system (CNS), this process is known as neuroinflammation and is characterized by the activation of the microglia and astrocytes population [1–3], the increase in concentration of different cytokines, and chemokines and, under certain conditions, the disruption of the blood brain barrier and the subsequent invasion of cells from the hematopoietic system to the injury site [4]. Thus, the burden of protecting CNS from injury falls on a specific group of cells: microglia, astrocytes, and mast cells. Mast cells can be found within the brain and their functions include the attractant and activation of other immune cells by secreting proinflammatory cytokines, and chemoattractants [5]. Astrocytes also contribute to the immune response by liberating both pro- and anti-inflammatory cytokines, chemokines and complement components [6]. Finally, microglia (CNS-resident macrophages) represent the main effector cells of the immune system in the CNS. Under physiological conditions, they stay as a quiescent population. In response to an infection or injury, they activate acquiring a reactive inflammatory phenotype characterized by an increased proliferation, morphological changes, and the release of several inflammatory molecules such as cytokines, reactive oxygen species, and nitric oxide [7]. 

Some aspects of the neuroinflammatory response result beneficial for CNS outcomes. Among these benefits, neuroprotection phenomena, the maintenance of neurogenesis as a mechanism of brain repair, the mobilization of neural precursors for repair, remyelination, and even axonal regeneration are included [8, 9]. However, neuroinflammation can be harmful too, leading to neuronal damage. Benefits and detriments balance depends largely on the magnitude of the immune response. In this sense, it is important to distinguish between the two types of responses in which inflammatory mechanism has traditionally been classified: acute and chronic inflammation. The first one comprises the immediate and early response to an injurious agent and is basically a defensive response that paves the way for repair of the damaged site being typically short-lived and unlikely to be detrimental to long-term neuronal survival [10]. The chronic response occurs when the harmful stimulus persists over time and contrary to the acute form, it is a long-standing and often self-perpetuating neuroinflammatory response which in the end, results in detrimental consequences for neurons [11]. Both acute and chronic responses have been related with neurodegenerative disorders. Thus, stroke and injury would associate with acute neuroinflammation while diseases such as multiple sclerosis or Alzheimer disease (AD) would be associated with the chronic form of the response. In this scenario, another important process commonly related with neurodegeneration and neuroinflammation emerges; the formation of new neurons in the adulthood or adult neurogenesis. 

Adult neurogenesis occurs in mammals principally in two brain regions: the subventricular zone (SVZ) and the subgranular zone (SGZ) of the hippocampus. The neuronal precursor cells (NPCs) that exist in both areas are a subset of astrocytes that give rise to intermediate progenitors which migrate and differentiate into new neurons of the hippocampus (in SGZ neurogenesis) or the olfactory bulb (in SVZ neurogenesis) [12]. Adult neurogenesis has been found to be altered in several neurodegenerative disorders such as Parkinson's disease, Huntington's disease, and AD (for a review, see [13]). On the other hand, neuroinflammation is a common feature of all these pathologies and, as it will be commented in detail later, it has also a relevant influence on adult neurogenesis. 

Due to the interconnection among these processes, it is important to consider them as a whole, taking into account that alterations affecting any of them would probably have consequences on the two others. 

In this review, we will summarize the role of neuroinflammation in both adult neurogenesis and Alzheimer disease. Related to this pathology, we will finally revise the recent advances concerning therapeutic approaches with neuroinflammatory mechanisms as a main target. 

## 2. Neuroinflammation and Adult Neurogenesis

It has been widely demonstrated that neuroinflammation affects adult neurogenesis having both detrimental and beneficial consequences which can result in enhancement and/or inhibition of the process. The final result depends largely on how microglia, macrophages, and/or astrocytes are activated and the duration of the inflammation [14]. Furthermore, the balance between the benefits and the detriments will have a profound impact on the efficiency of brain repair [15], which is of great importance in the context of the neurodegenerative disorders.

It is known that microglia, as the first immunological barrier against pathogens and environmental insults [16], exert the aforementioned dual effects on adult neurogenesis, resulting in pro or antineutogenic outcomes.

### 2.1. Proneurogenic Effects

An important study performed by Sierra and colleagues demonstrated that resting microglia play a crucial role in regulating the balance of newborn neurons in the hippocampus thanks to their phagocytic capacities [17]. Of the thousands of new cells born in the SGZ of the dentate gyrus, only a part of them differentiate and maturate into fully mature neurons. At least half of these cells die, probably through apoptosis, within the first few days to weeks after they are born [18–21]. Sierra et al. [17] provided data that attributed to microglia the function of removing those apoptotic new cells by phagocytosis. Furthermore, they importantly proved that this action did not require the activation of the microglial population. Recently, it has been reported that not only microglia are essential for adult neurogenesis but their functions and activity are importantly regulated by neuronal progenitor cells too. Thus, NPC-derived secreted factors are capable of modulating microglia activation, proliferation, and phagocytosis [22]. This crosstalk persists during neuron lifetime, since adult neurons are demonstrated to regulate microglia activation by constitutively expression of several neuroimmunoregulatory proteins such as CD200, CX_3_CL1 (or fractalkine), CD47, CD55, or HMGB1 (for a review see [23]). 

Further evidence of the proneurogenic effects of unchallenged microglia comes from the work of Walton and colleagues. By *in vitro* studies they showed that this population releases factors that rescue neuroblasts and instruct neuronal cell differentiation [24]. 

However, not only resting microglia exert benefits on adult neurogenesis. The acquisition of an active phenotype, under certain conditions, can be beneficial too [16], thanks to the liberation of anti-inflammatory cytokines with a broad range of actions on neurogenesis. Among these, we can highlight interleukin-4 and -10 (IL-4, IL-10) and transforming growth factor-beta (TGF-*β*). It is also important to keep in mind that cytokines classically considered as proinflammatory, such as interleukin-6 (IL-6), interleukin 1-*β* (IL-1*β*), and tumor necrosis factor-*α* (TNF-*α*) can be involved in the creation of a permissive environment for neurorepair too [25], as several studies have demonstrated (see Table 1).

Finally, not only cytokines derived from microglia can positively regulate neurogenesis. Other factors produced by immune system cells which are involved in the neuroinflammatory response have been shown to have certain influence. This is the case for granulocyte-macrophage colony stimulating factor (GM-CSF) [26] and the granulocyte-colony stimulating factor (G-CSF), with remarkable effects on the differentiation of NSC *in vitro* [27] (Table 1). 

### 2.2. Antineurogenic Effects

Neuroinflammation, although beneficial as a physiological response to maintain brain homeostasis, can have detrimental effects especially when it turns out to be a chronic response. Activated miroglia release proinflammatory cytokines which have been shown to affect largely neurogenesis. The aforementioned IL-6, TNF-*α*, and IL-1*β* but also interleukin-1-alpha (IL-1*α*), interleukin-18 (IL-18) and interferon-*γ* (IFN-*γ*) have detrimental consequences for proliferation and/or differentiation of NSC (Table 1). Among factors not released by microglia, CCL11 or eotaxin-1, a small chemokine known by its implication in allergic responses, has been recently linked to adult neurogenesis and ageing [28]. When administered systemically to young mice, eotaxin is able to impair neurogenesis producing as a consequence, learning and memory deficits [28]. Furthermore, it seems to affect directly the number and size of neurospheres formed from primary NPCs [28], suggesting that precursor cells probably have receptors capable of binding the cytokine.

Finally, it is especially noteworthy to keep in mind that most of the aforementioned factors are not only produced by microglia, but for astrocytes too, which contribute to the pathogenesis of neurodegenerative disorders as will be commented later. 

The effects of the abovementioned cytokines on neurogenesis are summarized in Table 1. 

## 3. Neuroinflammation and Alzheimer Disease

Neurodegenerative diseases are characterized by the progressive loss of neurons from specific regions of the CNS, which is believed to account for the cognitive and motor impairments suffered by patients with these neurodegenerative disorders. Importantly, inflammation is a process that has been closely related to the onset of many of these diseases, such as Amyotrophic Lateral Syndrome (ALS), Multiple Sclerosis (MS), Parkinsons Disease (PD) and Alzheimer Disease (AD) [29–33]. Indeed, aberrant inflammatory responses are believed to play a role in the etiology of these disorders.

At present, AD is the most common cause of dementia in the elderly. It is estimated that 27 million people are affected worldwide [34] and this number is expected to triple by 2050 due to the increase of the population life expectancy [35]. AD is a neurodegenerative disorder which affects brain regions that control memory and cognitive functions, which implies that patients finally lose their memory and ability to learn, to reason, to communicate and to carry out daily activities [36]. There are two different types of Alzheimer Disease, familiar Alzheimer Disease (FAD) and Sporadic Alzheimer Disease (SAD), and the origin of the disease could be different in both familial and sporadic cases. In terms of FAD, mutations in three different genes (presenilin-1 PS-1, presenilin 2 PS-2 and amyloid precursor protein APP) are likely to promote the onset of the disease whereas for SAD, different risk factors might be involved. Nevertheless, downstream the initial causes of the disease some common factors may be involved [37]. At a molecular level, AD is characterized by the presence of two main hystopathological hallmarks: senile plaques (extracellular aggregates composed by amyloid peptide or A*β*) and neurofibrillary tangles (intracellular aggregates composed by hyperphosphorylated forms of tau protein). A*β* results from the cleavage of APP and, although it seems to have important developmental functions in cell differentiation and possibly in the establishment of synapses [38, 39], its functions in adult brain still remain unclear. On the other hand, tau protein, the major component of neurofibrillary tangles, is a microtubule associated protein which contributes to the normal function of this intracellular support structure. Under pathogenic conditions, tau is highly phosphorylated reducing its ability to bind to microtubules [40] and favoring the formation of protein aggregates.

As a part of the inflammatory response, gliosis is a common feature of AD. Activated astrocytes and microglia are characteristically found in abundance near neurons and plaques. Besides, AD brains show increased expression of several pro-inflammatory cytokines which are hardly found in normal brains [61–64]. The main hypothesis proposes the chronic inflammatory reaction as a response to the accumulation of A*β* plaques and tangles [65]. Although initial inflammatory response can be beneficial, chronic activation of astrocytes and microglia has been shown to induce necrosis in adjacent neurons by releasing reactive oxygen intermediates, nitric oxide, proteolytic enzymes, complementary factors, or excitatory amino acids [66].

A*β* and their precursor APP are potent activators of glial cells [67, 68]. Thus, A*β* binds to the microglial cell surface regulating extracellular signal regulated kinase (ERK) and mitogen-activated protein kinase (MAPK) pathways which induces proinflammatory gene expression leading to cytokine and chemokine production [69]. Several chemokines and their receptors have been found to be upregulated in the AD brain. For example, macrophage inflammatory protein (MIP)-1*α* has been detected in reactive astrocytes nearby A*β* plaques [70]. In the same manner, changes in levels of many cytokines have been described not only in AD brains but also in blood and cerebrospinal fluid from patients. Thus, increased levels of IL-1*α*, IL-1*β*, IL-6, TNF-*α*, and GM-SF have been reported in brain tissue [71, 72]. In serum from patients, an increase in eotaxin, a cytokine recently linked to adult neurogenesis and ageing has been also detected [28] and, correlating to this, an increase in the expression of its receptor, CCR3, has been found in AD brains, especially in microglia [73]. Importantly, several works describe interactions between components of the senile plaques and cytokines, which could be generating a positive feedback loop for the neuroinflammatory process [74]. For example, A*β* protein is able to potentiate the secretion of IL-6 and IL-8 under several conditions [75]. Similarly, astrocytes might be activated by A*β* [76], contributing to generating a proinflammatory environment via the liberation of several cytokines and chemokines.

However, in some situations the role of microglia has been shown to be beneficial, since the activation of this population can decrease the accumulation of A*β* thanks to their phagocytic ability which facilitates the clearance and degradation of the aggregates [77]. Besides, microglia can be beneficial too through the secretion of growth factors such as the glia-derived neurotrophic factor (GDNF) which favors neuron survival [78]. Similarly, a relatively unknown cytokine, fractalkine, which has been demonstrated to have important neuroprotective characteristics, has been recently linked to the disease. Thus, fractalkine signaling (with its only receptor CX_3_CR1) has been found to be altered in AD brains in which reduced levels of the cytokine has been described [79].

Finally, it is noteworthy to keep in mind that although neurons have been traditionally believed to be passive bystanders in neuroinflammation, they seem to contribute to the production of neuroinflammatory molecules, a phenomenon that could be relevant in AD. Thus, the production of IL-1, IL-6, and TNF-*α* by neurons has been reported. Indeed, these neuronal chemokines act as messengers between neurons and glial cells (for a review, see [80]).

As neuroinflammation represents an important hallmark in AD and, as it has been shown in Section 2, it has a remarkable influence on adult neurogenesis, modulating the inflammatory environment could be beneficial not only for improving the deficits directly provoked by the disease but also for stimulating the endogenous ability of the brain for repairing the damage. In this sense, it is important to highlight that, especially in AD, understanding the role of adult neurogenesis is of great importance considering that one of the neurogenic zones is the hippocampus, structure responsible for cognitive and learning capacities which is largely affected in AD patients.

To date, it is not fully understood how adult neurogenesis is affected in neurodegenerative disorders. In AD, contradictory results have been obtained from the study of several animal models and the study of brain tissue by biochemical and histological approaches. Different effects on proliferation, differentiation, and survival have been reported in AD transgenic animal models with mutations in APP and tau or in both (for a review, see [81]). Besides, alterations affecting NPCs and differentiation of newborn neurons have been described in a glycogen-synthase kinase 3 overexpressing mouse model (GSK-3*β* has been proposed as a key protein in AD [82]), with an important role of microglia as a mediator of these damaging effects [83, 84] among which, morphology alterations of newborn neurons are included [85]. In humans, first data were obtained by Nagy and colleagues in 1997 [86]. In this pioneer work, the authors reported an increase in Ki-67 marker (staining proliferating cells) in the hippocampus from AD patients. In 2004, Jin et al. confirmed this result restricting it specifically to neurons [87]. However, in 2006, another group, although reported an increase in the proliferative status of presenile AD brains, they demonstrated that these precursors finally differentiated into glial cells [88]. 

Consequently, although adult neurogenesis remains an unknown field to be further explored in Alzheimer disease, it is likely to be affected in the disease. Taking into account that this process is known to contribute to learning and memory [89–91], an appropriate form to improve the subsequent deficits in cognitive functions associated to AD would result from modulating factors, such as those implicated in neuroinflammation, directly related to the correct formation of the newborn neurons. Finally, we cannot forget that adult neurogenesis declines with age, being a not so common event in the elderly [92], a fact that reinforces even more the idea of preserving or stimulating it as a brain repair mechanism.

## 4. Therapeutic Approaches

Based on the evidence that involves neuroinflammation in the pathogenesis of Alzheimer disease, researchers have focused their efforts on the development of anti-inflammatory drugs as a treatment option for patients with AD. Drugs such as the NSAIDs and glucocorticoid steroids have been studied.

### 4.1. NSAIDs

NSAIDs is the abbreviation for “nonsteroidal anti-inflammatory drugs.” They constitute a large family of compounds which includes the salicylate, propionic acid, acetic acid, fenamate, oxicam, and the COX-2 inhibitor classes (enzymes which regulate the homeostatic production of prostanoids, implicated in the inflammatory response) [36]. Epidemiological evidences indicate that NSAIDs may lower the risk of developing AD [93–95], since patients suffering from rheumatoid arthritis and osteoarthritis have been shown to inversely correlate with the risk of develop AD. Although beneficial effects have been observed both *in vitro* and *in vivo* (for a review, see [80]), unfortunately, clinical trials of NSAIDs in AD patients have not been very fruitful [96], especially in the case of COX-2 inhibitors. Thus, COX-2 inhibitor rofecoxib and the COX-1 and COX-2 inhibitor naproxen, were unable to slow the progression of the disease in patients with mild-moderate AD [97]. As a possible hypothesis, it could be postulated that NSAIDs might be useful to prevent the pathology but ineffective once the disease occurs.

### 4.2. Glucocorticoid Steroids

These compounds are considered as potent anti-inflammatory agents that modulate the transcription of several inflammatory molecules reducing, for example, the expression of proinflammatory cytokines and complement proteins [98]. However, the results obtained in AD patients have not been very promising. Thus, the use of some glucocorticoid steroids, such as prednisone, has not revealed any benefit in terms of slowing cognitive decline [99].

However, other therapies have been developed not directly directed to reduce inflammation but to the main targets that induce the chronic activation of these mechanisms, such as A*β* plaques or tau protein. 

### 4.3. A*β*-Based Immunization Strategies

the efficacy of these therapies has been demonstrated in mouse models of the disease. In 1999, Schenk and colleagues proved in an APP mutant mice that A*β*-directed vaccination prevented the development of neuritic A*β* plaques reducing them in older animals [100]. Furthermore, vaccination was effective in reducing age-dependent learning deficits which correlated with reductions in both soluble A*β* and tau [101]. Although APP model does not recapitulate all common features of AD, they resemble an early preclinical phase of the disease, which may be the optimal phase to initiate a therapy for preventing the disorder [102]. Importantly, efficacy of the vaccine was also found in a nonhuman primate, the Caribbean vervet [103]. Regarding the promising results obtained in animal models, a clinical trial was launched with AN-1792 containing preaggregated synthetic A*β*_42_ and the adjuvant QS-21 [104]. Although 6% of the patients developed meningoencephalitis, some others developed A*β*-antibody titres that correlated with a slow cognitive decline [105], and this result encouraged the development of several antibody fragments and humanized A*β*-specific antibodies, which are currently in various stages of clinical trials [102]. Time will tell whether these therapies are effective enough to halt the disease.

### 4.4. Tau-Based Immunization Approaches

First approach applying tau-based immunization was carried out by Rosenmann and colleagues in 2006 by injecting C57BL/6 wild-type animals with full-recombinant human tau. The experiments are unsuccessful since the vaccination caused encephalitis [106]. Subsequently, other groups tried active immunization approaches using tau phosphopeptides, obtaining promising results in tau transgenic models, in which they were able to prevent tau pathology in the absence of obvious side effects (for a review, see [107]). However, one of the main problems derived from these studies is the difficulty to translate them into clinical practice. This is due to the fact that the vaccinations were observed to prevent tau-related problems when administered prior to the appearance of any pathology or cognitive deficit, something that, nowadays, would be impossible regarding the current diagnosis methods. At present, the tau-targeted therapies that are in clinical trials target tau phosphorylation by GSK-3, microtubule stability, and aggregation [108].

Finally, it is important to highlight that considering that A*β* pathology depends on the presence of tau [109, 110] and that A*β* deposition is absent in many tauopathies (neurodegenerative diseases associated with the pathological aggregation of tau), it is absolutely necessary to pursue a tau-targeted treatment probably in combination with an A*β*-targeting approach.

## 5. Concluding Remarks

Although mechanisms underlaying Alzheimer disease remain unclear, neuroinflammation seems to be a common feature to neurodegenerative diseases with an important contribution to the pathology, affecting among others, physiological processes with a repairing function such as the adult neurogenesis process. Thus, modulating neuroinflammation by targeting causing agents or/and trying to ameliorate their harmful effects could be of great importance to possibly, prevent AD pathology and contribute to stimulate endogenous repairing mechanisms as the formation of new neurons. 

## Figures and Tables

**Table 1:** Effects of different cytokines on neurogenesis.

Cytokine	Effects on neurogenesis	References
IL-1*α*	Increased astrocyte lineage	[41]
IL-1*β*	Stimulation of NPCs proliferation and differentiation Decreased proliferation, survival, and neuronal differentiation Increased astrocyte differentiation	[42] [43] [44]
IL-4	Increased oligodendrogenesis	[45]
IL-6	Decreased proliferation, survival, and neuronal differentiation	[46] [47] [48] [49]
	Differentiation of NSC to neuronal lineages	
	Increased neurogenesis	
IL-10	Increased proliferation	[50] [51]
IL-18	Decreased survival Increased neuronal differentiation	[52]
IFN-*γ*	Decreased proliferation and survival of multipotent progenitors Promotion of differentiation and neurite outgrowth	[53] [54] [55]
CCL11 (eotaxin-1)	Decreased Sox-2 progenitors, proliferation, and neuronal differentiation	[28]
CX3CL1 (fractalkine)	Decreased neurogenesis	[56]
GM-CSF	Stimulation of NPCs differentiation	[57]
G-CSF	Promotes NPCs differentiation	[27]
TGF-*β*	Decreased proliferation Increased survival and neural differentiation	[58] [59] [60]
